# Adaptation of retinal ganglion cell function during flickering light in the mouse

**DOI:** 10.1038/s41598-019-54930-4

**Published:** 2019-12-05

**Authors:** Tsung-Han Chou, Jonathon Toft-Nielsen, Vittorio Porciatti

**Affiliations:** 1 Bascom Palmer Eye Institute, University of Miami Miller School of Medicine, Miami, Florida USA; 2 Intelligent Hearing Systems, Corp, Miami, Florida USA

**Keywords:** Predictive markers, Retina

## Abstract

Rapid dilation of retinal vessels in response to flickering light (functional hyperemia) is a well-known autoregulatory response driven by increased neural activity in the inner retina. Little is known about flicker-induced changes of activity of retinal neurons themselves. We non-invasively investigated flicker-induced changes of retinal ganglion cell (RGC) function in common inbred mouse strains using the pattern electroretinogram (PERG), a sensitive measure of RGC function. Flicker was superimposed on the pattern stimulus at frequencies that did not generate measurable flicker-ERG and alter the PERG response. Transition from flicker at 101 Hz (control) to flicker at 11 Hz (test) at constant mean luminance induced a slow reduction of PERG amplitude to a minimum (39% loss in C57BL/6J mice and 52% loss in DBA/2J mice) 4–5 minutes after 11 Hz flicker onset, followed by a slow recovery to baseline over 20 minutes. Results demonstrate that the magnitude and temporal dynamics of RGC response induced by flicker at 11 Hz can be non-invasively assessed with PERG in the mouse. This allows investigating the functional phenotype of different mouse strains as well as pathological changes in glaucoma and optic nerve disease. The non-contact flicker-PERG method opens the possibility of combined assessment of neural and vascular response dynamics.

## Introduction

Rapid dilation of retinal vessels in response to flickering light (functional hyperemia) is a well-known autoregulatory response^1–3^ that is believed to reflect an increase in blood flow to meet the increased metabolic demand of activated amacrine cells and ganglion cells (RGC) in the inner retina^1^. The link between increased neural activity and corresponding increase of blood flow has been much investigated^1,4–6^, although the neuro-vascular dynamics and the molecular mechanisms underlying functional hyperemia are not completely understood. A number of studies have investigated in different mammals the magnitude and temporal dynamics of flicker-induced changes of arterial diameter and blood flow^1,2^. Little is known about the magnitude and temporal dynamics of retinal neurons themselves in response to flicker. Here we non-invasively investigate flicker-induced changes of RGC function in common inbred mouse strains.

## Methods

### Animals and husbandry

In the present study, 13 C57BL/6J (B6) mice and 13 DBA/2J (D2) mice 4 months old purchased from Jackson Labs (Bar Harbor, ME, USA) were used. All procedures were performed in compliance with the Association for Research in Vision and Ophthalmology (ARVO) statement for use of animals in ophthalmic and vision research. The experimental protocol was approved by the Animal Care and Use Committee of the University of Miami. All mice were maintained in a cyclic light environment (12 h light: 50 lux – 12 h: dark) and fed with Grain Based Diet (Lab Diet: 500, Opti-diet, PMI Nutrition International, Inc., Brentwood, MO). All procedures and testing were performed under anesthesia by means of intraperitoneal injections (0.5–0.7 ml/kg) of a mixture of ketamine (42.8 mg/ml) and xylazine (8.6 mg/ml).

### Assessment of RGC function

**Figure 1:**
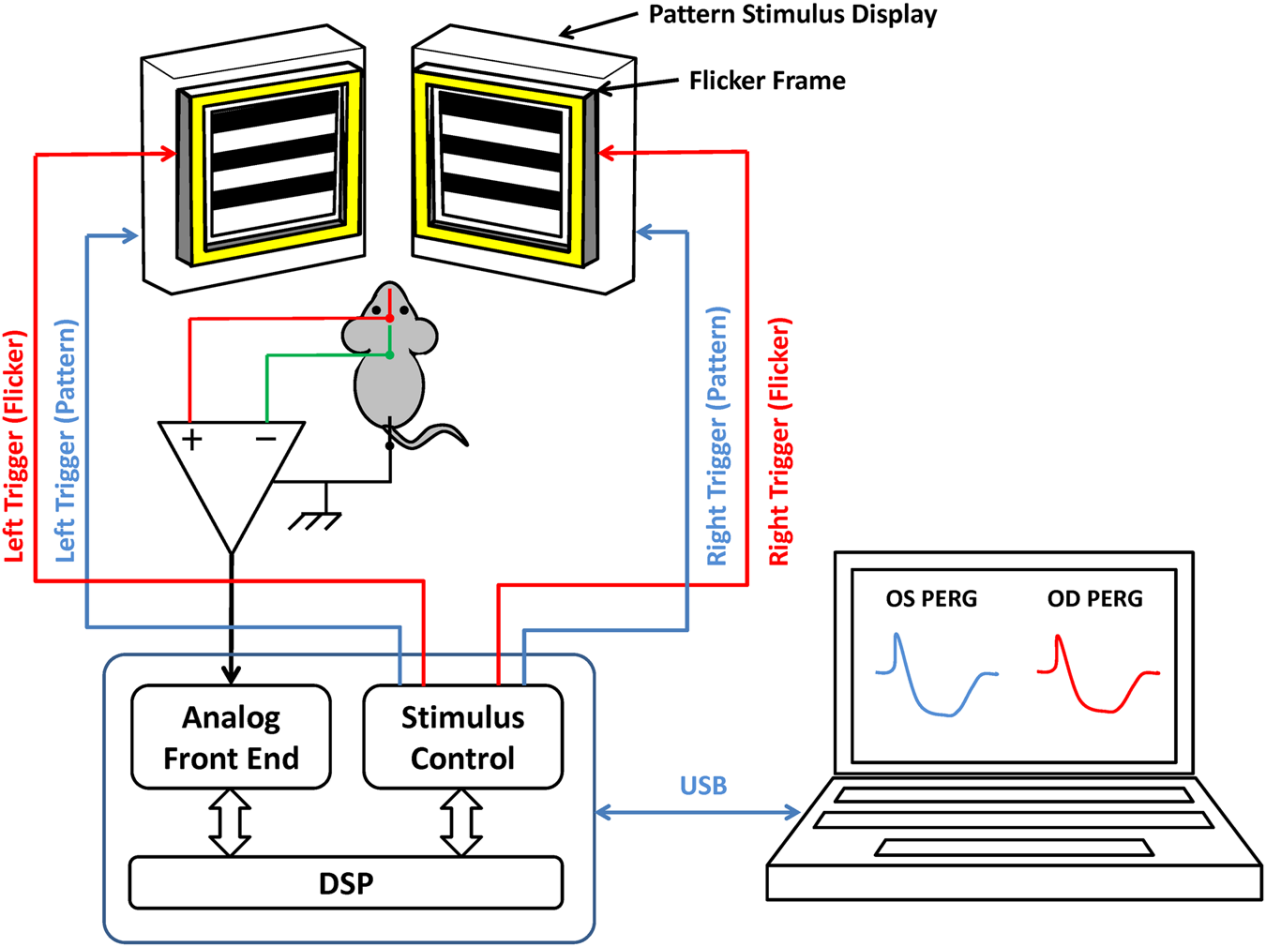
Block diagram for binocular PERG recording. Pattern stimuli are generated on two identical LED displays and presented separately to each eye. The control box generates independent TTL signals to trigger contrast reversal from each display at slightly different frequencies (right eye, 1.016 Hz; left eye 1.008 Hz) as well as TTL signals to trigger flickering light from non-patterned frames at different frequencies (101 Hz, 11 Hz). PERG signals are recorded continuously by means of subcutaneous needle electrodes (active, snout; reference, back of the head; ground, tail) and fed to one-channel acquisition system over 369.024 seconds (corresponding to 372 epochs). The PERG response for each eye is extracted from the acquired signal by synchronizing the averaging method with two noncorrelated frequencies (right eye, every 492 ms; left eye, every 496 ms). The responses are then displayed on the screen of the laptop that controls the stimulation/acquisition box.

**Figure 2:**
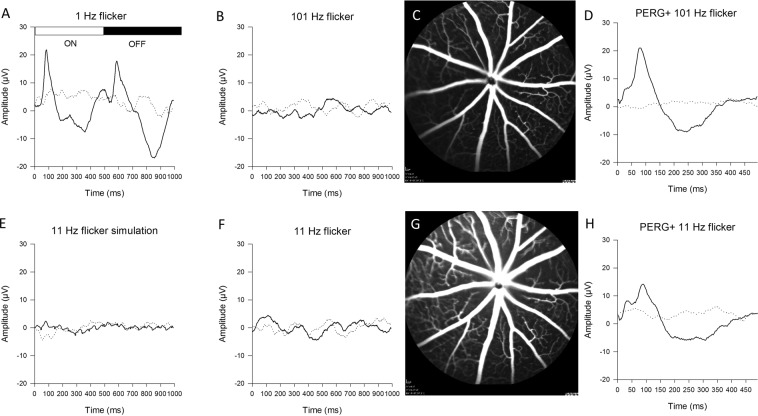
Representative examples of the effect of flicker on ERG, vascular dilation, and PERG. (**A**) 1 Hz Flicker (50% duty cycle) generates robust light-adapted ERG responses at both light onset and offset. (**B**) 101 Hz Flicker does not generate a signal distinguishable from noise (dotted line). (**F**) 11 Hz flicker also does not generate a measurable signal due to interference between onset and offset ERGs. (**E**) Simulation of interference between onset and offset ERGs by superimposing onset and offset ERGs shown in A at 11 Hz frequency. (**C**,**G**) Fluorescein angiography images obtained with superimposition of flicker at either 101 Hz (**C**) or 11 Hz (**G**). (**D,H**) PERG recorded with superimposition of flicker at either 101 Hz (**D**) or 11 Hz (**H**). In panels A,B,E,F, noise response were obtained by averaging even and odd epochs in counterphase. In panels (**D,H**) noise response were obtained with the pattern contrast set to zero (uniform mean luminance).

RGC function was assessed by Pattern Electroretinogram (PERG), an electrical signal that specifically depends on the presence of functional RGCs^7^ and is commonly used in human and experimental models of glaucoma and optic neuropathies^8,9^. The PERG technique for simultaneous recording from both eyes has been previously described in detail^10,11^. In the present study, we used a commercially available instrument for PERG recording (Jorvec Corp. FL, USA) modified to superimpose a flickering field to the patterned stimulus (Fig. 1). In brief, anesthetized mice were gently restrained in a holder allowing unobstructed vision and kept at a constant body temperature of 37.0 °C using feedback-controlled heating pad controlled by a rectal probe. Pupils were undilated and small (<1 mm), which insured a large depth of focus. PERG signals were recorded from a subcutaneous stainless steel needle (Grass, West Warwick, RI, USA) placed in the snout. As the bioelectric field of PERG is orthogonal to the eye axis^12^ while that of the luminance ERG is coaxial^13^, PERG and 1 Hz ON-OFF ERG have approximately similar amplitudes using snout recording (see examples in Fig. 2A,D). The reference and ground electrodes were similar needles placed medially on the back of the head and at the root of the tail, respectively. Visual stimuli were presented at each eye independently from 10 cm distance and consisted of contrast-reversal of gratings (0.05 cycles/deg, 98% contrast) generated on two light-emitting diode (LED) tablets (15 × 15 cm square field, 800 cd/m² mean luminance) alternating at slightly different frequencies around 1 Hz (OD, 1.016 Hz; OS, 1.008 Hz). Independent PERG signals from each eye were retrieved using one channel continuous acquisition and phase-locking average over 372 epochs for each eye. PERG amplitude was measured peak-to-trough using a software that automatically detected the positive peak and the negative trough in the PERG waveform (typically the P1 peak to the N2 trough). Noise responses were obtained by computing the difference between even and odd epochs^14^. The patterned LED display was surrounded by a LED square frame (internal size 15 × 15 cm, external size 18 × 18 cm) flickering light. The flickering frame had the same mean luminance of the patterned field, and could be modulated (square-wave, 50% duty cycle) at either 101 Hz or 11 Hz at constant mean luminance. These frequencies were asynchronous with the pattern reversal frequencies. At 101 Hz, flickering light could not be perceived by human observers.

### Experimental protocol

All experiments have been conducted under photopic conditions at constant mean luminance in order to remove the confounding factor of light adaptation^15^. The only variables were the temporal frequency of square-wave flicker (1 Hz vs. 101 Hz vs. 11 Hz) and that of pattern reversal (static vs. reversing). To study the effect of flicker per se, the flicker frame was modulated, and the pattern was static. As shown in Fig. 2A, 1 Hz flicker elicited a robust ERG at both stimulus ON and OFF. At 101 Hz (Fig. 2B), the ERG response was at noise level as expected, as this frequency was beyond the temporal resolution of mouse cone ERG^16^. At 11 Hz (Fig. 2F), the ERG was also much reduced. This required an explanation, as 11 Hz is well within the temporal window of the mouse cone ERG^16^. The reduced flicker ERG at 11 Hz was due to interference between positive and negative components of ON and OFF responses resulting in cancellation. This was demonstrated by superimposing ON and OFF responses shown in Fig. 2A with a time shift of 91.6 ms (1,008 ms ÷ 11 Hz). The simulated waveform (Fig. 2E) was in the noise range (compare in Fig. 2E the simulated 11 Hz flicker ERG and the noise). Flicker at 11 Hz, however, elicited a robust vascular response^17^ compared to 101 Hz flicker (compare fluorescein angiography images of Fig. 2C,G).

To study the effect of flicker on PERG, both the flicker frame and the patterned field were modulated. As the 11 Hz flicker ERG was in the noise range, the PERG signal was not expected to be visibly distorted by superimposition of flicker ERG. Any residual flicker ERG component on PERG was expected to be canceled out during PERG recording, as it was not synchronized with PERG averaging. To demonstrate this, we recorded a PERG with superimposed flicker at either 101 Hz or 11 Hz using a pattern stimulus with either 100% contrast or 0%  contrast (uniform field). As shown in Fig. 2D,H, the PERG at 0% contrast was at noise level, and the PERG at 100% contrast had no visible distortions due to flicker ERG contamination. Overall, results of this experiment provide the necessary framework for studying the effect of flicker on PERG, using 101 Hz as control and 11 Hz as test.

Four flicker + PERG responses were recorded in sequence: PERG + flicker 101 Hz (baseline), PERG + flicker 11 Hz (test) repeated 2 times, PERG + flicker 101 Hz (recovery). As each PERG recording (average of 372 epochs) required 6.25 min, the total recording time was 25 minutes. To better understand the temporal dynamics of PERG + flicker 11 Hz changes, PERG responses (average of 372 epochs each) were split in 12 sequential subaverages (31 epochs each) resulting in 48 sequential samples of 0.52 min each over the total recording time of 25 minutes. PERG + superimposed flicker responses were recorded from each eye^10^. As the results were similar in the two eyes, the results of the left eye only will be presented.

### Fluorescein angiography

As a demonstration that 11 Hz flicker generated a hemodynamic response, two mice for each group underwent fluorescein angiography (FA) images of retinal fundus. FA images were obtained using a scanning laser ophthalmoscope (Heidelberg retina tomograph LWP 542, excitation wavelength 488 nm). FA images were taken 15–20 minutes after I.P. injection of 40 µL of fluorescein 100 mg/ml to reach a stable level of fluorescence intensity in retinal vessels. FA images were taken while a panel of flickering LEDs was manually presented to the eye with an angle of approximately 45 degrees with the optical path. LEDs were flickering at either 101 Hz (baseline) or 11 Hz (test). As shown in Fig. 2C,G, 11 Hz flicker induced a noticeable vasodilation compared to 101 Hz flicker.

### Statistical analysis

To analyze flicker-induced PERG changes over time (PERG #1,2,3,4), the method of Generalized Estimating Equations (GEE) was used (IBM SPSS statistics Ver. 26). GEE is an unbiased non-parametric method to analyze longitudinal correlated data. In the analysis, PERG amplitude was the dependent variable and test period (PERG #1,2,3,4) and strain (B6, D2) were predictor variables. Main effects (test period, strain) and interaction between test period and strain were computed, as well as pairwise combinations between period and strain.

## Results

**Figure 3:**
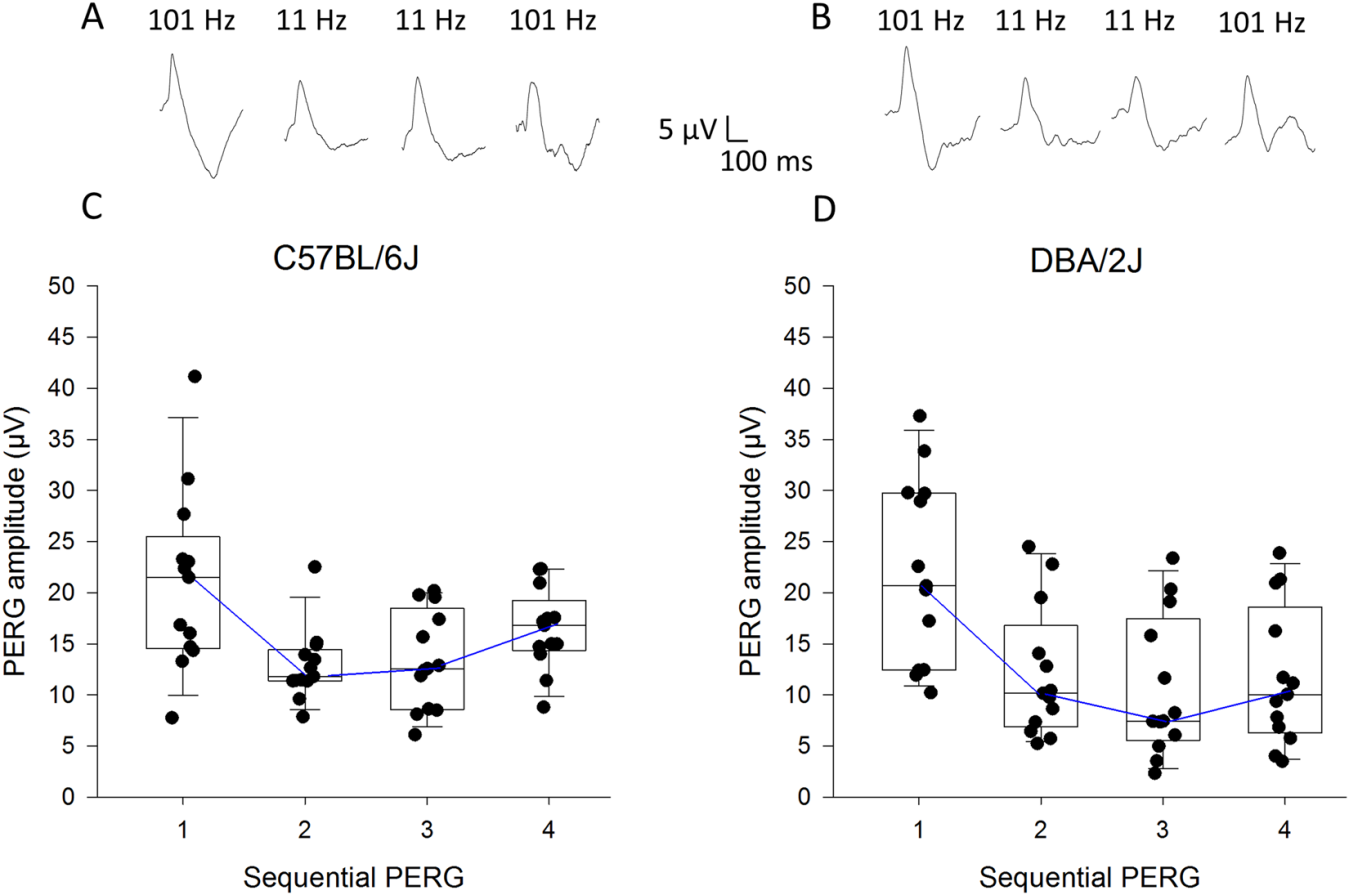
Effect of flicker on PERG in different mouse strains. (**A,B**) Representative examples of PERGs sequentially recorded in C57BL/6J mice (**A**) or DBA/2J mice (**B**) with superimposition of flicker at either 101 Hz or 11 Hz. (**C,D**) Distribution of PERG amplitudes sequentially recorded in individual mice (**C)**, C57BL/6J; (**D)**, DBA/2J with superimposition of flicker at either 101 Hz or 11 Hz.

Examples of PERG waveforms with superimposed flicker at either 101 Hz or 11 Hz recorded in B6 and D2 mice are shown in Fig. 3A,B, respectively. Corresponding distributions of peak-to-trough amplitudes are shown in Fig. 3C,D. In B6 mice, the median baseline PERG#1 amplitude was 21 µV and dropped to 12.9 µV (−39%) upon switching the flicker frequency from 101 Hz to 11 Hz (PERG#2). The PERG amplitude tended to recover baseline range in successive PERG #3 (11 Hz flicker) and PERG #4 (recovery, 101 Hz flicker). In D2 mice, flicker-induced PERG changes were similar, although the major drop in PERG amplitude upon transition to 101 Hz flicker to 11 Hz flicker occurred later at PERG#3, and was of larger magnitude (−52%); at PERG #4, the PERG amplitude was still well below baseline level. Generalized Estimating Equation (GEE) statistics for longitudinal data using PERG amplitude as dependent variable and test period (PERG #1,2,3,4) and strain (B6, D2) as predictor variables revealed a strong effect of period (P < 0.0001), no significant effect of strain (P = 0.35), but a strong interaction between period and strain (P < 0.001). Pairwise comparisons between period and strain revealed significant (P < 0.05) differences in PERG amplitude: B6 mice, PERG#1 > PERG#2, PERG#3, PERG#4; PERG#2 < PERG4; PERG#3 < PERG#4; D2 mice, PERG#1 > PERG#2, PERG#3, PERG#4. As a control that the results were not biased by progressive decrease of amplitude with time due to unrelated factors such anesthesia level, we recorded in different B6 mice and D2 mice (N = 4 for each group) sequential PERGs with constant superimposition of 101 Hz flicker. In both B6 and D2 mice, the PERG amplitude progressively decreased on average by 1.3 µV at each PERG recording (GEE statistics, P < 0.05), with no difference between B6 and D2 mice. Adjusting PERG amplitude data for progressive non-specific loss did not substantially change the results shown in Fig. 3, with the exception that in B6 mice PERG#4 was no longer different from PERG#1.

**Figure 4:**
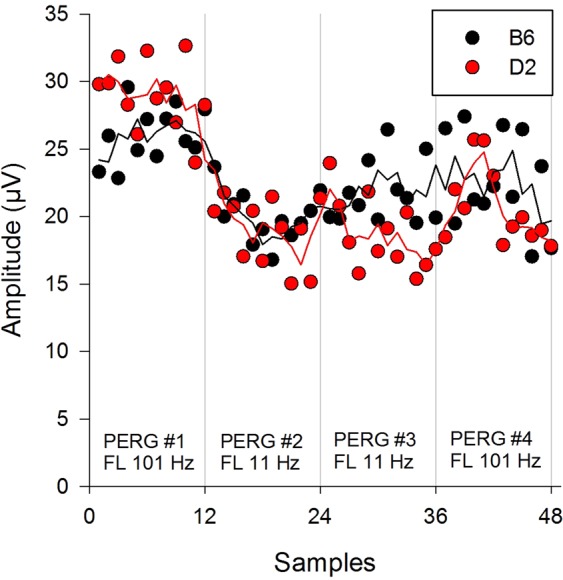
Time course of PERG amplitude change upon superimposition of flicker at either 101 Hz or 11 Hz in C57BL/6J mice and DBA/2J mice. Each sequential PERG (average of 372 epochs) was sampled off-line in 12 subaverages of 31 epochs of 0.52 minutes for a total of 48 samples (25 minutes). Each data point represents the group average of C57BL/6J mice (N = 13) and DBA/2J mice (N = 13). To facilitate visualization of time-dependent changes, data were smoothed by running average of three contiguous data points (continuous lines).

In order to better appreciate the time course of flicker-induced PERG changes, each of the four successive PERG responses in individual mice was processed off-line to split it in 12 consecutive samples 0.52 min each (48 consecutive samples over total recoding time of 25 minutes). Figure 4 shows the mean amplitude for each sample over time. Despite considerable variability due to limited averaging of 31 epochs per sample, it was possible to appreciate the temporal dynamics of PERG amplitude in both B6 and D2 mice. In control conditions (101 Hz flicker, samples 1–12) the PERG amplitude of both B6 and D2 mice was relatively stable. Upon test condition (11 Hz flicker, samples 13–36), the PERG amplitude of B6 mice progressively declined to reach a local minimum of at sample #19–20 (~4 min after 11 Hz flicker onset), and then slowly increased. In the recovery condition (samples 37–48), the PERG amplitude was in the range of baseline values. In D2 mice, the drop in PERG amplitude upon flicker appeared larger, a broad minimum occurred at sample # 21–22 (~5 min after 11 Hz flicker onset), and the PERG amplitude did not recover baseline values during the recovery condition (101 Hz flicker, samples 37–48).

## Discussion

Many studies have investigated the magnitude and temporal dynamics of flicker-induced functional hyperemia in the retina^1–3^. Models of functional hyperemia^1,18^ generally include two main mechanisms: neurovascular coupling, which relates increased neuronal activity to vascular dilation, and neurometabolic coupling, which relates increased neuronal activity to higher metabolic demands^19^. Better understanding of flicker-induced neurovascular/neurometabolic coupling in the retina requires quantification of the temporal dynamics of inner retinal neurons themselves. If flicker-induced neural activity substantially changes over time, then this will be inevitably be reflected on the temporal dynamics of neurovascular/neurometabolic coupling.

The temporal dynamics of murine RGC activity in response to flicker is not known. Here we show that the functional response of RGC in mice in response to flicker, as assessed by PERG, was not stationary. By switching the flicker frequency from 101 Hz to 11 Hz, the PERG amplitude progressively decreased (by 39% in B6 mice and by 52% in D2 mice), and then tended to slowly recover 101 Hz baseline values. The time course of flicker-induced PERG changes was slow, reaching a minimum after about 4 minutes in B6 mice and 5 minutes in D2. Several controls excluded that the flicker effect was artifactual or due to spurious effects. All experiments were done at constant mean luminance, the only variable being the frequency of flicker. While we cannot completely exclude a flicker effect in the outer retina, a rod contribution seems unlikely, as the mean luminance was well above the saturation level of rods^20^.

The stimulus and the recording conditions were designed in such a way as not to generate measurable flicker-ERG, which could have distorted the PERG waveform. The peak-to-trough PERG amplitude was measured in an automated manner. Data analysis was conducted to account for nonspecific changes of PERG amplitude over time.

The magnitude of flicker-induced reduction of PERG amplitude differed to some extent between young adult B6 and D2 strains, D2 mice displaying relatively larger reduction over time compared to B6. The reason for this difference may be due to strain differences in the number of RGCs^21^ and related difference in inner retinal circuitry^22^. Older D2 mice are known to be prone to glaucomatous RGC death^23^ that can be rescued with treatments supporting mitochondrial function^8^. As glaucoma progression in individual mice is variable, an altered PERG response to flicker may identify susceptible individuals and help predict development of disease. Testing this hypothesis was beyond the scope of this study.

The experimental set up with non-corneal recording electrodes was designed to avoid interference with the eye optics. As non-invasive dynamic retinal vessel analysis is now possible^17^, it is conceivable that an instrument can be built that allows simultaneous assessment of flicker-induced neural and vascular dynamics under identical conditions. This would allow a better insight on the neurovascular interactions in health and disease.

Flicker-induced PERG changes we have described in mice likely have a counterpart in human. Typically, patterned stimuli for steady-state PERG recording are generated by flickering black and white elements of the pattern in counterphase at 8 Hz, resulting in pattern contrast reversal at 16 Hz at constant mean luminance. Flicker-ERGs at 8 Hz elicited by individual black and white elements of the pattern are not recordable as they are in opposition of phase and are canceled out, leaving a 16 Hz contrast-reversal PERG response. Counterphase flickering patterns generate a strong hemodynamic response at the optic nerve head^2^, which is associated with slow decline of PERG to a plateau after about 2 minutes (adaptation)^24^. The magnitude of steady-state PERG adaptation is of the order of 30% in healthy subjects^25^ and is reduced in early glaucoma^26^ and optic neuritis^27^. As the human PERG is also recordable from non-corneal electrodes^28^, combined assessment of flicker-induced neural and vascular dynamics is possible by adapting available imaging instruments.

## Conclusions

Transition from flicker at 101 Hz to flicker at 11 Hz at constant mean luminance induces reduction of the RGC functional response, whose magnitude and temporal dynamics can be non-invasively assessed with PERG in the mouse. This provides a means for investigating the functional phenotype of different mouse strains as well as pathological changes in glaucoma and optic nerve disease. The non-contact flicker-PERG method opens the possibility of combined assessment of neural and vascular response dynamics.

## References

[1] Newman EA (2013). Functional hyperemia and mechanisms of neurovascular coupling in the retinal vasculature. J Cereb Blood Flow Metab.

[2] Riva CE, Logean E, Falsini B (2005). Visually evoked hemodynamical response and assessment of neurovascular coupling in the optic nerve and retina. Prog Retin Eye Res.

[3] Wanek J, Teng PY, Albers J, Blair NP, Shahidi M (2011). Inner retinal metabolic rate of oxygen by oxygen tension and blood flow imaging in rat. Biomed Opt Express.

[4] Hosford PS, Gourine AV (2019). What is the key mediator of the neurovascular coupling response?. Neurosci Biobehav Rev.

[5] Huneau C, Benali H, Chabriat H (2015). Investigating Human Neurovascular Coupling Using Functional Neuroimaging: A Critical Review of Dynamic Models. Front Neurosci.

[6] Iadecola C (2017). The Neurovascular Unit Coming of Age: A Journey through Neurovascular Coupling in Health and Disease. Neuron.

[7] Porciatti V (2015). Electrophysiological assessment of retinal ganglion cell function. Exp Eye Res.

[8] Williams PA (2017). Vitamin B3 modulates mitochondrial vulnerability and prevents glaucoma in aged mice. Science.

[9] Yu H, Porciatti V, Lewin A, Hauswirth W, Guy J (2018). Longterm Reversal of Severe Visual Loss by Mitochondrial Gene Transfer in a Mouse Model of Leber Hereditary Optic Neuropathy. Sci Rep.

[10] Chou TH, Bohorquez J, Toft-Nielsen J, Ozdamar O, Porciatti V (2014). Robust mouse pattern electroretinograms derived simultaneously from each eye using a common snout electrode. Invest Ophthalmol Vis Sci.

[11] Chou TH, Toft-Nielsen J, Porciatti V (2018). High-Throughput Binocular Pattern Electroretinograms in the Mouse. Methods Mol Biol.

[12] Chou TH, Porciatti V (2012). The bioelectric field of the pattern electroretinogram in the mouse. Invest Ophthalmol Vis Sci.

[13] Doslak MJ, Plonsey R, Thomas CW (1981). Numerical solution of the bioelectric field of the e.r.g. Med Biol Eng Comput.

[14] Schimmel H (1967). The (+) reference: accuracy of estimated mean components in average response studies. Science.

[15] Peachey NS, Goto Y, al-Ubaidi MR, Naash MI (1993). Properties of the mouse cone-mediated electroretinogram during light adaptation. Neurosci Lett.

[16] Krishna VR, Alexander KR, Peachey NS (2002). Temporal properties of the mouse cone electroretinogram. J Neurophysiol.

[17] Albanna W (2018). Non-invasive evaluation of neurovascular coupling in the murine retina by dynamic retinal vessel analysis. PLoS One.

[18] Nippert AR, Biesecker KR, Newman EA (2018). Mechanisms Mediating Functional Hyperemia in the Brain. Neuroscientist.

[19] Shu CY, Sanganahalli BG, Coman D, Herman P, Hyder F (2016). New horizons in neurometabolic and neurovascular coupling from calibrated fMRI. Prog Brain Res.

[20] Lyubarsky AL, Daniele LL, Pugh EN (2004). From candelas to photoisomerizations in the mouse eye by rhodopsin bleaching *in situ* and the light-rearing dependence of the major components of the mouse ERG. Vision Res.

[21] Williams RW, Strom RC, Rice DS, Goldowitz D (1996). Genetic and environmental control of variation in retinal ganglion cell number in mice. J Neurosci.

[22] Porciatti V, Chou TH, Feuer WJ (2010). C57BL/6J, DBA/2J, and DBA/2. J.Gpnmb mice have different visual signal processing in the inner retina. Mol Vis.

[23] Libby RT (2005). Inherited glaucoma in DBA/2J mice: pertinent disease features for studying the neurodegeneration. Vis Neurosci.

[24] Porciatti V, Sorokac N, Buchser W (2005). Habituation of retinal ganglion cell activity in response to steady state pattern visual stimuli in normal subjects. Invest Ophthalmol Vis Sci.

[25] Monsalve P (2018). Steady-state PERG adaptation: a conspicuous component of response variability with clinical significance. Doc Ophthalmol.

[26] Porciatti V (2014). Adaptation of the steady-state PERG in early glaucoma. J Glaucoma.

[27] Fadda A (2013). Reduced habituation of the retinal ganglion cell response to sustained pattern stimulation in multiple sclerosis patients. Clin Neurophysiol.

[28] Monsalve P (2017). Next Generation PERG Method: Expanding the Response Dynamic Range and Capturing Response Adaptation. Transl Vis Sci Technol.

